# Cold Wet Sheet Pack in Scarlatina

**Published:** 1880-06-20

**Authors:** Dudley M. Culver

**Affiliations:** Whitesville, Indiana


### COLD WET SHEET PACK IN SCARLATINA.

BY DUDLEY M. CULVER, M.D., OF WHITESVILLE, INDIANA.

We have just passed through a severe epidemic of scarlet fever in this section, and as we have had several deaths, it gave us room for scientific research in order to give our patients the best medical aid that was known to the profession. The epidemic at this point took a peculiar course, affecting adults more seriously than little children, and we had the disease in every form, from the most simple to the most malignant.

Our first cases we treated with antizymotic remedies, together with sponge baths and "old bacon grease" as external remedies, and despite our efforts we lost some cases; all this time fearing to use heroic treatment, on account of public opinion. But I have since formed a resolution to use such remedies hereafter as my judgment dictates, and let public opinion take care of itself. The cases we lost died, beyond a doubt, from uræmic poisoning, and we know that uræmic poisoning is caused by the rapid disintegration of tissue, induced by *excessive high temperature*, converting urea into carbonate of ammonia. Hence, this being the case, common sense ought to teach us that the excessive temperature in scarlet fever must be reduced or death will be the result. But what is an excessive temperature? I will say that in our research we found that if the temperature can be kept at or below 102° there is little danger, but in all of our malignant cases the temperature commenced at from 103½° to 105½°, and this is what we found to be excessive and truly dangerous; and truthfully does Prof. Reamy, of Cincinnati, speak when he says that "where the temperature stays at 105° for 24 consecutive hours he has never seen a case get well." Hence we see, and have found by experience, that the temperature is what we have to fight in the first five days, and guard against albuminuria after convalescence.

And they may suggest antizymotics, such as "sulphurous acid dilute, hyposulphite of soda, iron, etc.," but I find it is a dangerous practice in a disease as treacherous as malignant scarlet fever. There is no mistaking the fact that the temperature is what we have to fight, and, consequently, our main reliance must be on remedies that will reduce the temperature, and, at the same time, use diuretics as a shield against suppression of the urine.

Public opinion was strong against the *cold wet sheet pack* when it was first mentioned, the people supposing it would repel the eruption, drive the fever internally, and cause instant death; and as death was to be feared in malignant scarlet fever, we hesitated using such a heroic remedy, knowing too well that should our patients die the world would be ready to say that " the wet pack done it."

But finally I had a malignant case that was almost hopeless, Lelia S——, aged 12 years, temperature commenced at 103½° and rapidly rose to 105°. I could reduce the temperature by ordinary methods as low as 102°, but it would suddenly fly up again. She was restless, inclination to delirium, and threatened with uræmic poisoning. Finally I sent for my counselling physician, R. French Stone, M.D., of Bainbridge, an excellent physician of wide repute, and by our united efforts secured the right to use the cold wet sheet pack on this case.

Commenced Thursday evening at six o'clock with a temperature of 104½°. Stripped her of all clothing and wrapped her in a sheet wrung out of cold well-water. The result was very flattering. The temperature next morning at 7 a.m. was down to 101¼°, a reduction of 3¼° in thirteen hours. The wet sheet was renewed as often as it became warm.

This case made a good recovery, and we have tried the *wet pack* in several malignant cases since where the temperature would reach 105°, and with the same result. We used internally at the same time :

℞ Tinc. digitalis.......................................... ʒij.

Fl. ext. jaborandii...................................... ℥j.

Dose, a teaspoonful every four hours.

I will say that in place of repelling the eruption the pack brought it out more abundantly; and it is surprising how rapidly the patient becomes quieted and sleepy after the first application of the pack.

This is only necessary, understand me, in malignant cases, with high temperature. We don't treat all cases of fever alike, but treat a disease as the disease is presented to us. Many cases of scarlet fever will yield at once to diuretics and diaphoretics. Such cases, of course, need no wet sheet. But in conclusion let me say, *Fight the temperature as a consuming fire.—Ind. Practitioner.*

